# Paediatric healthcare in Manhiça district through a gender lens: a retrospective analysis of 17 years of morbidity and demographic surveillance data

**DOI:** 10.7189/jogh.15.04010

**Published:** 2025-02-21

**Authors:** Núria Balanza, Aura Hunguana, Sara Ajanovic, Rosauro Varo, Justina Bramugy, Teodimiro Matsena, Tacilta Nhampossa, Dan Ouchi, Arsénio Nhacolo, Jéssica Dalsuco, Antonio Sitoe, Llorenç Quintó, Sozinho Acácio, Ariel Nhacolo, Maria Maixenchs, Khátia Munguambe, Inácio Mandomando, Pedro Aide, Francisco Saúte, Caterina Guinovart, Charfudin Sacoor, Quique Bassat

**Affiliations:** 1 Barcelona Institute for Global Health (ISGlobal), Barcelona, Spain; 2 Facultat de Medicina i Ciències de la Salut, Universitat de Barcelona (UB), Barcelona, Spain; 3 Centro de Investigação em Saúde de Manhiça (CISM), Maputo, Mozambique; 4 Instituto Nacional de Saúde, Ministério da Saúde, Marracuene, Mozambique; 5 Institució Catalana de Recerca i Estudis Avançats (ICREA), Barcelona, Spain; 6 Paediatrics Department, Hospital Sant Joan de Déu (University of Barcelona), Barcelona, Spain; 7 CIBER de Epidemiología y Salud Pública, Instituto de Salud Carlos III, Madrid, Spain

## Abstract

**Background:**

Sex and gender are important determinants of health. Gender-based health inequities in the paediatric population have been reported in various countries, but data remain limited. In Mozambique, research on this topic is very scarce. Here we aimed to explore whether boys and girls in Manhiça district, southern Mozambique, differ in access to and provision of healthcare.

**Methods:**

This retrospective analysis includes data on all paediatric (<15 years old) visits to six outpatient clinics and admissions to one hospital in Manhiça district from 2004 to 2020, collected through the morbidity surveillance system of the Manhiça Health and Demographic Surveillance System (HDSS). We compared characteristics and outcomes between boys and girls using descriptive statistics, standardised mean differences, and logistic regression. Post-discharge events were analysed using Cox proportional hazards regression and Fine-Gray competing risk regression. Minimum community-based incidence rates of outpatient clinic visits and hospitalisations were calculated using demographic surveillance data from the Manhiça HDSS and analysed with negative binomial regression.

**Results:**

Girls represented 49.2% (560 630 out of 1 139 962) of paediatric visits to outpatient clinics and 45.1% (18 625 out of 41 278) of hospitalisations. The girls-to-boys incidence rate ratio (IRR) for hospitalisations was 0.81 (95% confidence interval (CI) = 0.79–0.84). Both boys and girls experienced symptoms for a median duration of one day (interquartile range (IQR) = 1–2) before seeking care. Severe manifestations at presentation to an outpatient clinic or upon hospitalisation tended to be less frequent in girls (girls-to-boys odds ratios (ORs) = 0.71–1.11). Girls were less frequently referred or admitted to hospital after an outpatient clinic visit (OR = 0.82; 95% CI = 0.79–0.86 and OR = 0.85; 95% CI = 0.84–0.87, respectively). The hospital case fatality ratio was 4.1% in boys and 4.2% in girls. The median duration of hospitalisation was three days (IQR = 2–5) and did not differ between boys and girls. Revisits to outpatient clinics, hospital readmissions, and hospital post-discharge mortality were similar in both groups.

**Conclusions:**

Girls had fewer referrals and admissions to hospital in Manhiça district, but they were also less likely to present with severe manifestations. Other studied indicators of healthcare access and provision were overall similar for boys and girls. Further research is needed to continue assessing potential gender biases and sex differences in paediatric healthcare in Mozambique.

Sex and gender are major drivers of health and well-being. Sex, defined as the biological and physiological differences between males, females, and intersex people, can influence disease susceptibility and progression. Gender refers to the socially constructed norms, roles, and behaviours for men/boys, women/girls, and other gender identities, which affects other determinants of health, health risk behaviours, and access to and quality of healthcare [1]. Understanding the impact of sex-specific differences and gender inequities on health is crucial for informing interventions and policies aimed at improving health outcomes and leaving no one behind. However, research in this area has been predominantly focused on adults, with limited attention given to the paediatric population.

Male infants are at a higher risk of death than their female counterparts during the first months of life [2], which is generally attributed to biological differences that make them more vulnerable to congenital diseases, perinatal illnesses, and infectious diseases [3–6]. Yet this survival advantage in female children might be lost or even reversed in settings where girls are at an increased social risk. A few studies have investigated whether gender-driven inequities exist in children’s care and feeding practices, access to health services, timely seeking of healthcare, and management by healthcare providers [7]. Most of this research comes from Asia, where some studies have described that girls face disadvantages compared to boys which might be explained by societal norms and family or cultural practices [7–10]. Studies on this topic including data from sub-Saharan African countries are very scarce. Some have reported that girls are admitted to hospital less frequently, have higher in-hospital mortality, or that their caregivers seek healthcare for them less often, but others did not find significant differences, with findings varying by study and country [11–13].

Mozambique ranked 118 out of 166 countries in the Gender Inequality Index in 2022, indicating remarkable inequalities between women and men in health, empowerment, and labour market [14]. Nevertheless, there is a lack of data on the impact of gender on health among Mozambique’s paediatric population. Only two multi-country reports assessed healthcare-seeking behaviour for children under five in Mozambique using data from the Demographic and Health Surveys programme. According to these, caregivers took boys and girls to a healthcare provider with similar frequency in the presence of diarrhoea, fever, or suspected pneumonia [11,12]. Some studies have suggested an excess of female under-five mortality in Mozambique, but interpretation of findings is complex and results vary depending on the methodology used and the specific years analysed, with no such excess observed in older children [15–20]. Therefore, further country-specific research is needed to understand potential sex- and gender-based differences in health during childhood and early adolescence.

This study aimed to explore whether there are differences in access to and provision of healthcare between boys and girls younger than 15 years in the district of Manhiça, southern Mozambique. We compared different indicators using surveillance data from the Manhiça Health and Demographic Surveillance System (HDSS) of the Manhiça Health Research Centre (*Centro de Investigação em Saúde de Manhiça* (CISM)) during a 17-year period (2004–2020).

## METHODS

### Study site

The district of Manhiça is located in Maputo province, southern Mozambique, 80 km north of the capital city of Maputo [21,22]. With ~ 209 000 inhabitants in 2022 and an area of 2380 km^2^ on a plain crossed by the Incomati river [23], it is the paradigm of a poor, resource-limited rural sub-Saharan setting. The population is predominantly young and there is a sharp reduction of males from 20 years of age and above. Most residents are engaged in small businesses, practice subsistence farming, or work in agricultural companies. A more detailed description of the study area and population can be found elsewhere [21–23].

The prevalence of human immunodeficiency virus (HIV) in Manhiça is among the highest in the world, with an adult community prevalence of around 30–40% and an HIV vertical transmission rate that has decreased over the years [24–27]. *Plasmodium falciparum* malaria transmission is perennial in the area and peaks during the rainy season (November–April), but an upscale of malaria control tools in the last two decades has contributed to a notable decrease in malaria cases [28]. Major causes of paediatric morbidity and mortality include malaria, pneumonia, HIV/acquired immunodeficiency syndrome, diarrheal diseases, and malnutrition [29].

The district of Manhiça has 21 healthcare facilities, which include the Manhiça District Hospital (MDH), a rural hospital in Xinavane, and several outpatient clinics [23]. The MDH is the main healthcare facility in the area, used for primary healthcare by nearby population and one of the two referral health centres for Manhiça district. It has a 110-bed inpatient ward, an outpatient clinic, a maternal and child health clinic with a small surgical room, a round-the-clock emergency department, and basic laboratory facilities. Children visiting the outpatient clinic at MDH can be admitted to the paediatric ward of the hospital, immediately or after being under observation for some hours in a day-care unit. Severely ill patients admitted to MDH can be transferred to Maputo Central Hospital, a tertiary healthcare facility in the capital city of Maputo. Outpatient clinics have basic medical equipment and limited supplies. They offer primary healthcare services and not inpatient care, but they may have a few beds available for observation and post-delivery. All clinical management and treatment are free of charge, except for a standard subsidised fee for the outpatient medication to be taken at home.

### The Manhiça HDSS

The CISM has run an HDSS in the district of Manhiça since 1996. It comprises both a demographic surveillance system covering the general population and a morbidity surveillance system monitoring paediatric patients attending healthcare facilities in the study area [21,22].

#### Demographic surveillance

The catchment area of the demographic surveillance gradually expanded since its creation to capture the entire district of Manhiça, with the largest increase occurring in 2014. Each person living within the HDSS surveillance area is issued a unique HDSS permanent identification number. Households are visited twice a year to collect sociodemographic information at the individual and household levels, including data on births, deaths, and migrations. This information is supplemented by daily hospital visits, weekly updates from key community informants, and, more recently, notifications via a toll-free call centre that operates 24 hours a day, seven days a week, 365 days a year. Data was collected on paper forms and double-entered until 2011, after which electronic collection was implemented. Information validation rules, input constraints, and error-checking scripts are established to ensure data quality.

#### Paediatric morbidity surveillance

Seven healthcare facilities in Manhiça district are currently involved in monitoring paediatric morbidity. The number of participating facilities has increased over the years in line with the demographic surveillance area expansions. Each time a child under 15 years attends one of these healthcare facilities, a standardised paper-based form is completed with the information about the health event (a two-page form for outpatient clinic visits and a four-page form for hospital admissions). These include demographic data, signs and symptoms upon presentation, basic laboratory investigations, multiple diagnoses, treatments prescribed, and outcomes. The HDSS permanent identification number of the patients is also collected in these questionnaires, enabling demographic and illness events to be linked for children residing within the Manhiça HDSS study area. Data are collected using paper questionnaires and double-entered. Similarly to the demographic surveillance system, data quality control measures are established during and after data entry.

### Study design

In this retrospective analysis, we included all visits by children younger than 15 years to the outpatient clinics of MDH, Maragra, Malavele, Palmeira, Taninga, and Ilha Josina Machel, as well as all hospitalisations of children younger than 15 years at MDH, from 1 January 2004 to 31 December 2020 (Figure S1 in the **Online Supplementary Document**). The Taninga outpatient clinic was incorporated into the morbidity surveillance system in 2005, while the Palmeira and Malavele clinics were added in 2009. The period 2004–2020 corresponds to a timeframe during which the same standardised questionnaires have been used in the morbidity surveillance system. We compared various indicators reflecting access to and provision of healthcare between boys and girls. These included percentages and minimum community-based incidence rates (MCBIRs) of outpatient clinic visits and hospitalisations, duration of symptoms before visiting an outpatient clinic and before hospitalisation, presence of severe manifestations at presentation to an outpatient clinic and upon hospitalisation, referrals or admissions to hospital, hospital case fatality ratios (CFRs), time from hospitalisation to in-hospital death, length of hospital stay, revisits to outpatient clinics within seven days, hospital readmissions within 28 days, post-discharge mortality within 28 days, and paediatric mortality rates in the Manhiça HDSS study area. Demographic surveillance data were used to identify paediatric patients residing in the area, estimate population denominators, and retrieve additional mortality data for deaths occurring outside the hospital. The databases used from the Manhiça HDSS included male and female sex data for children, but did not provide information on intersex biological status or gender identities. In the context of this study, male children were considered boys and female children were considered girls, and are indicated as such throughout the text, in line with our focus on understanding gender as a social determinant of health.

### Data analysis

Descriptive data were reported as counts (%), median (interquartile range (IQR)), or mean (standard deviation). Continuous variables were compared between two groups using standardised mean differences [30]. Univariable logistic regression models were fitted to estimate girls-to-boys odds ratios (ORs) of presenting with severe manifestations or having certain outcomes. An OR greater than one denotes the odds are greater in girls. Definitions used for severe manifestations can be found in Table S1 in the **Online Supplementary Document****.** For outcomes, discharge home after an outpatient clinic visit or after hospitalisation was used as the reference category. We also calculated CFRs during hospitalisation, excluding patients who absconded or were transferred to the Maputo Central Hospital.

The MCBIRs of outpatient clinic visits and hospitalisations were calculated by dividing the number of events among children residing in the Manhiça HDSS study area (i.e. those with an HDSS permanent identification number in the clinical questionnaire) by the total time at risk for the paediatric population of Manhiça residents inferred from demographic surveillance data. Children did not contribute to the numerator or denominator when they were living outside the study area. The periods of hospitalisation at MDH were also excluded from the time at risk. For girls-to-boys incidence rate ratios (IRR), we used mixed-effects negative binomial regression models, with individual as a random effect and no adjustment for other covariates.

We studied revisits to outpatient clinics within seven days, hospital readmissions within 28 days, and hospital post-discharge mortality within 28 days in children living in the Manhiça HDSS study area. For revisits and readmissions, we defined index visits/admissions as those in which children were either discharged home or absconded. We studied time to the first-or-only event within the determined time at risk (recurrent events occurred in <0.1% of children). After the follow-up period (seven or 28 days) any subsequent visit or admission was considered unrelated to a previous one and modelled as a new index visit/admission. Fine-Gray competing risk regression models, considering death as a competing event, were used to estimate girls-to-boys subdistribution hazard ratios (SHRs) and cumulative incidence functions of revisits and readmissions [31]. For hospital post-discharge mortality, we defined index hospitalisations as those with a known outcome different from in-hospital death. We used a single-discharge approach, considering the first hospital admission as reference and excluding subsequent readmissions within the following 28 days, consistent with previous analyses in Manhiça district [32]. Cox proportional hazards regression models were used to calculate girls-to-boys hazard ratios (HRs) of post-discharge mortality, and cumulative incidences were estimated as 1 - Kaplan-Meier survival function. Deaths occurring outside the healthcare facilities were retrieved from demographic surveillance data. In all analyses of post-discharge outcomes, children who migrated outside the Manhiça HDSS study area before the end of the follow-up period were censored at that moment. Standard errors were adjusted for within-child correlation, but the models did not include additional covariates.

Mortality rates for boys and girls in Manhiça district were calculated using demographic surveillance data. The number of infants, under-5, and under-15 deaths in each calendar year was divided by the total number of live births that occurred in the same year, expressed per thousand live births.

We conducted all data analyses both on the complete set and by stratifying it into four age groups: <1 year, 1–4 years, 5–9 years, and 10–14 years. Most variables had a small percentage of missing values and those were excluded when computing the different statistics. The statistical software Stata version 16.1 (StataCorp, College Station, TX, USA, 2019) and *R* version 4.2 or above (R Core Team, Vienna, Austria, 2022) were used.

### Ethical approval

The CISM’s HDSS was approved by the local administrative authorities (government of Manhiça district and president of Manhiça municipality), by the Institutional Ethics Review Board, and by the National Committee for Bioethics in Health of Mozambique. All residents of the Manhiça HDSS study area gave signed individual informed consent to be enrolled in the ongoing HDSS. For resident children, parents or guardians gave signed informed consent and children assented if aged 12–17 years.

## RESULTS

### Study population characteristics

Over the study period, from January 2004 to December 2020, there were 1 147 471 across the six outpatient clinics and 41 354 admissions to MDH by children younger than 15 years. Of these, 1 147 441 (99.9%) and 41 278 (99.8%) had available data on the child’s sex, respectively. A total of 7479 visits to the outpatient clinic at MDH were identified as direct referrals from the other five outpatient clinics. To avoid double counting these same events, only the first available contact with the healthcare system was retained, resulting in a final sample size of 1 139 962 paediatric outpatient clinic visits. The total number of outpatient clinic visits and hospitalisations by calendar year and age group can be found in Figure S2 in the **Online Supplementary Document**.

The median age of children visiting an outpatient clinic was 3.4 years (IQR = 1.4–7.3), with a median age in boys of 3.3 years (IQR = 1.3–7.0) and in girls of 3.6 years (IQR = 1.4–7.7). Among outpatient clinic visits, the most frequent diagnoses were upper respiratory tract infections (32.1% for boys and 33.5% for girls) and malaria (24.5% for boys and 24.4% for girls). In children admitted to MDH, the median age was 1.6 years (IQR = 0.7–3.3), the same for boys and girls. The most frequent diagnoses in inpatients included malaria (43.0% for boys and 45.0% for girls), lower respiratory tract infections (27.0% for boys and 26.4% for girls), and gastrointestinal diseases (13.8% for boys and 12.4% for girls).

### Frequency of outpatient clinic visits and hospitalisations

**Figure 1:**
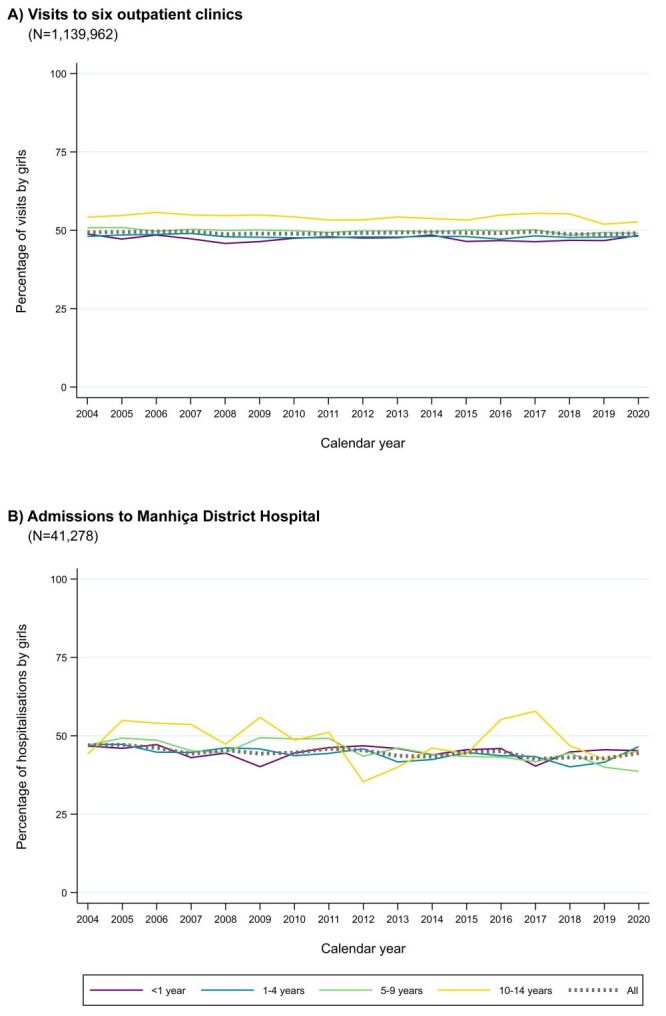
Percentage of paediatric outpatient clinic visits and hospitalisations by girls from 2004–2020 in Manhiça district.

Girls accounted for 49.2% (560 630 out of 1 139 962) of the total outpatient clinic visits. With respect to admissions to MDH, girls represented 45.1% (18 625 out of 41 278). These overall percentages were constant over time, with no marked downward or upward trend during the 17 years of the study. Results remained similar across age groups, except for children aged 10–14 years. In this oldest group, girls consistently accounted for a higher percentage of outpatient clinic visits, and there was more variability in the percentage of hospitalisations by girls over time (**Figure 1**).

When restricting the analysis to children living in the Manhiça HDSS study area, the overall MCBIRs of outpatient clinic visits and hospitalisations were lower in girls compared to boys (Table S2 in the **Online Supplementary Document**). The overall girls-to-boys IRR for outpatient clinic visits was 0.97 (95% CI = 0.96–0.98). This ratio remained consistent across age groups, except for children aged 10–14 years, where the MCBIR for outpatient clinic visits was higher for girls and the IRR was 1.09 (95% CI = 1.07–1.11). The overall girls-to-boys IRR for hospitalisation was 0.81 (95% CI = 0.79–0.84), consistent when stratifying by age group (Table S2 in the **Online Supplementary Document**).

### Duration of symptoms before visiting an outpatient clinic and before hospitalisation

Caregivers were enquired about the number of days that their child had experienced fever, cough, diarrhoea, or vomiting during the current disease before visiting an outpatient clinic or being admitted to MDH. Both boys and girls had any of these symptoms for a median duration of one day (IQR = 1–2) before visiting an outpatient clinic, and a median duration of two days (IQR = 1–3) before hospitalisation. For each symptom individually, the median duration was also the same for boys and girls. Results were consistent when stratified by age group, with all standardised mean differences <0.1 (**Table 1**).

**Table 1:** Reported symptoms in children and their duration before visiting an outpatient clinic and before hospitalisation in Manhiça district*

		N	Any symptom	Fever		Cough		Diarrhoea		Vomiting	
			**Median days (IQR)**	**%**	**Median days (IQR)**	**%**	**Median days (IQR)**	**%**	**Median days (IQR)**	**%**	**Median days (IQR)**
**Visits to six outpatient clinics**											
All	Boys	579 332	1 (1–2)	73.1	1 (1–2)	52.6	2 (1–3)	7.7	1 (1–1)	7.5	1 (1–1)
	Girls	560 630	1 (1–2)	74.2	1 (1–2)	53.4	2 (1–3)	6.9	1 (1–1)	7.3	1 (1–1)
*<1 y*	Boys	110 343	1 (1–2)	62.1	1 (1–2)	67.8	2 (1–3)	15.9	1 (1–1)	9.5	1 (1–1)
	Girls	99 158	1 (1–2)	60.6	1 (1–2)	67.8	2 (1–3)	14.4	1 (1–1)	9.0	1 (1–1)
*1–4 y*	Boys	256 752	1 (1–2)	73.8	1 (1–2)	57.3	2 (1–3)	8.8	1 (1–1)	7.6	1 (1–1)
	Girls	236 980	1 (1–2)	74.1	1 (1–2)	59.2	2 (1–3)	8.2	1 (1–1)	7.5	1 (1–1)
*5–9 y*	Boys	138 130	1 (1–2)	78.5	1 (1–2)	42.2	2 (1–3)	2.0	1 (1–1)	6.6	1 (1–1)
	Girls	137 064	1 (1–2)	80.4	1 (1–2)	44.7	2 (1–3)	2.1	1 (1–1)	6.9	1 (1–1)
*10–14 y*	Boys	74 107	1 (1–2)	77.4	1 (1–2)	32.9	2 (1–3)	2.3	1 (1–1)	5.5	1 (1–1)
	Girls	87 428	1 (1–2)	80.7	1 (1–2)	35.2	2 (1–2)	2.4	1 (1–1)	5.5	1 (1–1)
**Admissions to Manhiça District Hospital**											
All	Boys	22 653	2 (1–3)	84.7	1 (1–3)	58.5	2 (1–3)	20.6	1 (1–2)	22.2	1 (1–2)
	Girls	18 625	2 (1–3)	85.5	1 (1–3)	58.8	2 (1–3)	19.1	1 (1–2)	22.7	1 (1–2)
*<1 y*	Boys	8 096	2 (1–3)	77.4	1 (1–3)	62.6	2 (1–3)	28.5	1 (1–2)	24.5	1 (1–2)
	Girls	6 619	2 (1–3)	78.9	1 (1–3)	61.7	2 (1–3)	25.8	1 (1–3)	23.6	1 (1–2)
*1–4 y*	Boys	11 271	2 (1–3)	89.7	1 (1–3)	59.9	2 (1–3)	18.4	1 (1–2)	20.8	1 (1–1)
	Girls	9 203	2 (1–3)	89.8	2 (1–3)	60.6	2 (1–3)	17.7	1 (1–2)	21.0	1 (1–1)
*5–9 y*	Boys	2 516	2 (1–3)	87.2	1 (1–3)	44.4	2 (1–4)	8.1	1 (1–2)	21.0	1 (1–1)
	Girls	2 105	2 (1–3)	89.7	2 (1–3)	47.4	2 (1–4)	7.5	1 (1–2)	25.0	1 (1–2)
*10–14 y*	Boys	770	2 (1–3)	79.7	2 (1–3)	40.4	3 (2–6)	10.3	1 (1–3)	22.4	1 (1–1)
	Girls	698	2 (1–3)	80.1	2 (1–3)	41.1	3 (1–7)	9.8	1 (1–2)	31.0	1 (1–2)

IQR – interquartile range.

*The median (IQR) number of prior days with any symptom was calculated for all children who visited an outpatient clinic or were hospitalised. The median (IQR) number of prior days for each specific symptom was calculated for children who reported experiencing that symptom. All standardised mean differences in the duration of symptoms between boys and girls are <0.1. Missing data in all variables is <1%.

### Presence of severe manifestations at presentation to an outpatient clinic and upon hospitalisation

**Figure 2:**
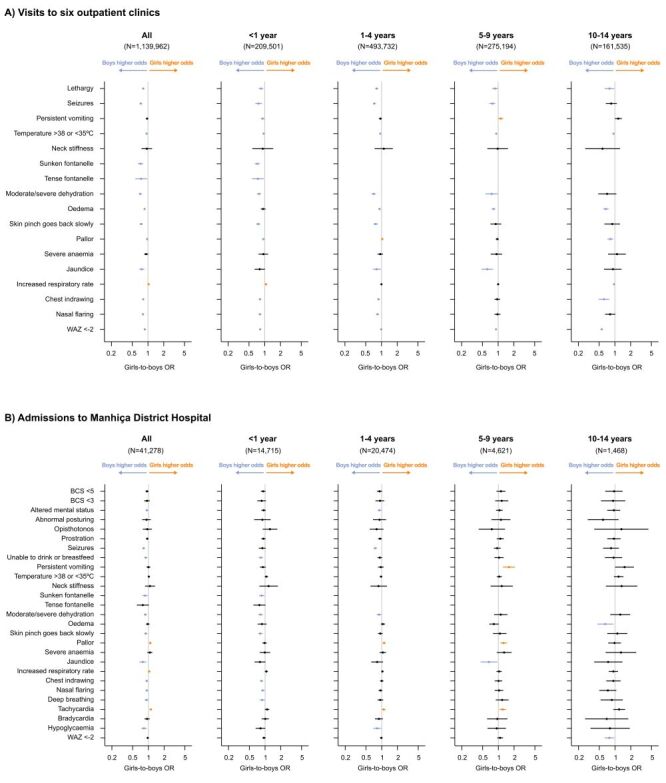
Girls-to-boys odds ratios of severe manifestations at presentation to an outpatient clinic and upon hospitalisation in Manhiça district. Odds ratios and 95% CIs from univariable logistic regression models are shown on a logarithmic scale. Points to the right indicate that girls have higher odds than boys of having a certain severe manifestation when visiting an outpatient clinic or being admitted to Manhiça District Hospital, whereas points to the left indicate that boys have higher odds than girls of having it. Estimates are highlighted in orange or blue if the 95% CIs do not cross one. Definitions used for severe manifestations can be found in Table S1 in the **Online Supplementary Document**. Sunken or tense fontanelle was calculated only in children aged <1 year. Missing data in all variables is <5%, except for sunken/tense fontanelle (5% in outpatient clinic visits, 8% in hospitalisations), severe anaemia (45% in outpatient clinic visits, 9% in hospitalisations), and hypoglycaemia (9% in hospitalisations). BCS – Blantyre Coma Scale, CI – confidence interval, OR – odds ratio, WAZ – weight-for-age z-score.

We selected 17 severe general manifestations among children visiting outpatient clinics and 27 among hospitalised children. When visiting an outpatient clinic, girls had lower odds for 13 out of 17 severe manifestations (girls-to-boys OR = 0.71–0.96). For instance, these include dehydration (OR = 0.71; 95% CI = 0.67–0.76), seizures (OR = 0.73; 95% CI = 0.69–0.77), and jaundice (OR = 0.76; 95% CI = 0.68–0.85). Girls only had higher odds for increased respiratory rate (OR = 1.03; 95% CI = 1.02–1.04) (**Figure 2**, Panel A). Upon admission to MDH, severe manifestations were more common. Girls had lower odds for 11 out of 27 severe manifestations (girls-to-boys OR = 0.78–0.93). However, ORs were closer to one compared to those for same manifestations observed at presentation to an outpatient clinic. The odds of three out of 27 severe manifestations were higher in girls: increased respiratory rate (OR = 1.04; 95% CI = 1.00–1.08), pallor (OR = 1.09; 95% CI = 1.03–1.14), and tachycardia (OR = 1.11; 95% CI = 1.06–1.17) (**Figure 2**, Panel B). All ORs for the entire cohort and each age group are presented in **Figure 2**. Nevertheless, the differences between boys and girls in the percentages of patients with severe manifestations were small in absolute terms (Figure S3 in the **Online Supplementary Document**).

### Patient management and outcomes of outpatient clinic visits and hospitalisations

**Figure 3:**
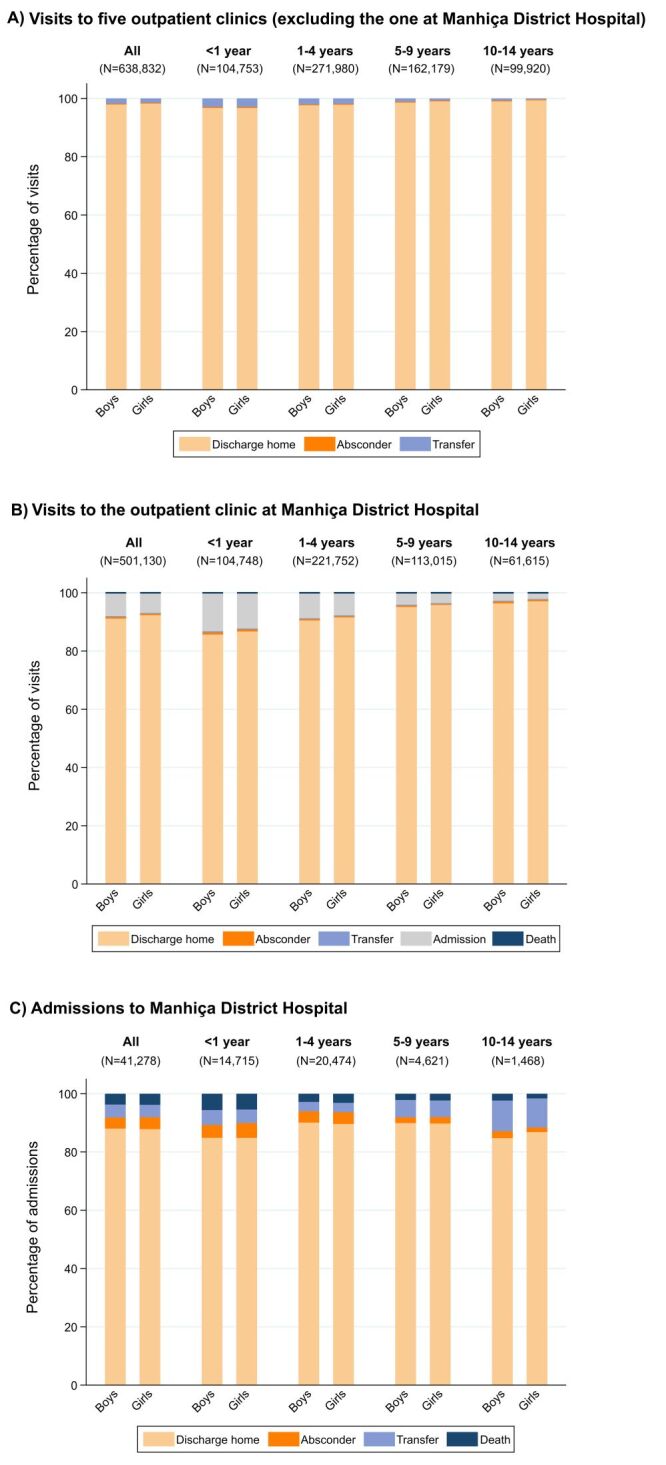
Outcomes of paediatric outpatient clinic visits and hospitalisations in Manhiça district. Outcomes of visits to the outpatient clinic at Manhiça District Hospital (**Panel B**) were calculated separately from those of visits to the other five outpatient clinics (**Panel A**), as possible outcomes differ. Outcomes of visits to the outpatient clinic at Manhiça District Hospital (**Panel B**) are a combination of the outcomes that occurred immediately after the clinical visit or after being under observation for some hours in a day-care unit of Manhiça District Hospital. Missing data in outcomes is <1%.

In all outpatient clinics other than the one at MDH, 1.8% of boys and 1.5% of girls were referred to a higher-level healthcare facility (usually to MDH) after a clinical evaluation (girls-to-boys OR = 0.82; 95% CI = 0.79–0.86). In children who visited directly the MDH outpatient clinic, a decision for admission to MDH was reached in 8.0% of boys and 6.9% of girls (girls-to-boys OR = 0.85; 95% CI = 0.84–0.87). Similar results were obtained when stratifying by age group, with smaller ORs in older age groups (Table S3 in the **Online Supplementary Document**). Other possible outcomes at the outpatient clinic level (i.e. absconder, transfer from the MDH outpatient clinic to Maputo Central Hospital, or death) were uncommon in both boys and girls (**Figure 3****,** Panels A–B; Table S3 in the **Online Supplementary Document**).

Upon admission to MDH, the median length of hospital stay did not differ between boys and girls (three days (IQR = 2–5) for each group), regardless of stratification by age group or restriction to only children who were finally discharged home (Table S4 in the **Online Supplementary Document**). An explorative breakdown of diagnostic tests conducted and treatments given in boys and girls can be found in Table S5 in the **Online Supplementary Document**. The hospital CFR was 4.1% in boys and 4.2% in girls (girls-to-boys OR = 1.02; 95% CI = 0.92–1.13). Similarly, there were no significant differences in terms of absconders or transfers to Maputo Central Hospital. This was also observed when stratifying by age group (**Figure 3****,** Panel C; Table S3 in the **Online Supplementary Document**). Time to in-hospital death was similar overall in boys and girls, with both groups having a median of two days (IQR = 1–5) from hospitalisation to in-hospital death (Table S6 in the **Online Supplementary Document**).

### Post-discharge outcomes after an outpatient clinic visit and after hospitalisation

**Figure 4:**
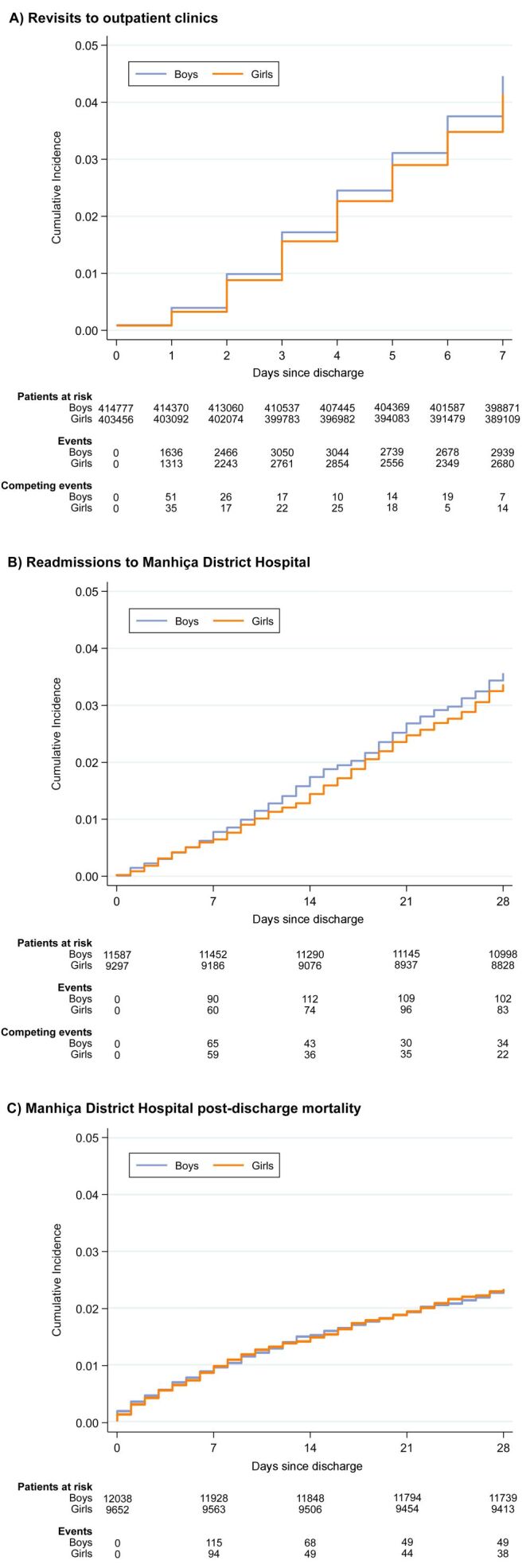
Cumulative incidence of post-discharge outcomes in children after an outpatient clinic visit and after hospitalisation in Manhiça district. For revisits to outpatient clinics (**Panel A**) and hospital readmissions (**Panel B**), index visits or hospitalisations were defined as those in which children were either discharged home or absconded, and death was treated as a competing event. For hospital post-discharge mortality, index hospitalisations were defined as those with a known outcome different from in-hospital death.

Within seven days after visiting an outpatient clinic and being either discharged home or absconding, 4.5% (18 552 out of 414 777) of boys and 4.2% (16 756 out of 403 456) of girls revisited an outpatient clinic. The girls-to-boys SHR of revisiting was 0.93 (95% CI = 0.90–0.95) (**Figure 4****,** Panel A). Within 28 days after leaving the MDH due to being discharged home or absconding, 3.6% (413 out of 11 587) of boys and 3.4% (313 out of 9297) of girls were readmitted. The subdistribution hazard rates of hospital readmission were not different in the two groups (girls-to-boys SHR = 0.94; 95% CI = 0.81–1.10) (**Figure 4****,** Panel B). Using the first admission to MDH as reference and excluding subsequent readmissions within the next 28 days, 2.3% (281 out of 12 038) of boys and 2.3% (225 out of 9652) of girls died within 28 days after hospital discharge. The hazard rates of post-discharge mortality did not differ between boys and girls (girls-to-boys HR = 1.00; 95% CI = 0.84–1.19) (**Figure 4****,** Panel C). Results by age groups were similar and can be found in Table S7 and Figures S4–S6 in **the ****Online Supplementary Document**.

In addition, at the population level, the infant, under-5, and under-15 mortality rates in the Manhiça HDSS study area over the study period were slightly higher for boys than girls (Figure S7 in the **Online Supplementary Document**).

## DISCUSSION

In this paediatric cohort from southern Mozambique, comprising over 1.1 million visits to outpatient clinics and 41 000 hospitalisations from 2004 to 2020, we found no overall major differences between boys and girls in most studied indicators of access to and provision of healthcare. However, results indicate that girls were referred and admitted to hospital less frequently, and they were also less likely to present with severe manifestations. By using comprehensive morbidity and demographic surveillance data spanning 17 years we were able to assess multiple healthcare aspects separately for boys and girls, expanding our analysis beyond the healthcare facility level to encompass post-discharge outcomes and provide additional insights at the community level.

Gender bias by caregivers favouring boys over girls could manifest in differences in access to healthcare, with girls being taken to healthcare facilities less frequently, at later stages of illness, and with more severe symptoms. In our study population, girls accounted for 49.2% of visits to outpatient clinics and the girls-to-boys IRR of visits at the population level was 0.97 (95% CI = 0.96–0.98). Frequency of utilisation of healthcare services is difficult to interpret in our study, as we do not know how many children did not seek care but needed it. However, our results suggest no important differences regarding healthcare-seeking behaviour according to referred days with symptoms before visiting an outpatient clinic. This is in line with the two previous reports using Demographic and Health Surveys data from Mozambique, which showed no difference in the proportion of boys and girls with symptoms of common illnesses who were taken to a healthcare provider. One study reported a boys-to-girls healthcare-seeking ratio of 1.02 (95% CI = 0.93–1.10), while the other reported no significant differences in percentages [11,12]. In other sub-Saharan African countries, the limited studies comparing healthcare utilisation and time to seek healthcare between boys and girls when they face illness have usually shown either no significant differences or suggest an advantage for boys [11,12,33–42]. In our study, we also found that severe manifestations at presentation to an outpatient clinic were generally more frequently observed in boys. However, since these differences do not seem to be related to delays in seeking healthcare, they could be attributed to variations in how symptoms are assessed or to sex-based differences in pathophysiological mechanisms. Boys might be more susceptible to severe disease and rapid progression due to biological factors, such as having weaker immune systems during infancy [6]. In addition, we did not observe differences in the frequency of absconding decisions between boys and girls.

Gender-based inequities in the care provided by healthcare workers may lead to differences in clinical decisions and patient outcomes. Especially in resource-limited settings, with scarce laboratory and imaging services, the assessment of severity and prediction of adverse outcomes in paediatric conditions rely largely on symptom and sign-oriented clinical guidelines. This may introduce subjectivity, with the potential for sociocultural influences to affect the evaluation process and introduce a gender bias. If boys are favoured, girls may be less frequently referred or admitted to hospital, experience longer hospital stays due to complications, or have higher in-hospital mortality. In our study population, a slightly higher percentage of boys than girls (1.8 vs. 1.5%) were referred to a higher-level healthcare facility from outpatient clinics or were hospitalised (8.0 vs. 6.9%) if directly visiting the MDH outpatient clinic. This led to girls accounting for 45.1% of the hospital admissions and a girls-to-boys IRR for hospitalisations of 0.81 (95% CI = 0.79–0.84). Once hospitalised, the length of hospital stay, CFR, and time to in-hospital death were similar for boys and girls. Previous studies in children with pneumonia, diarrhoea, or fever have also shown that girls tend to be admitted to hospital less frequently than boys, but their CFRs are similar or higher in girls [12,13,42]. Regarding post-discharge outcomes (i.e. revisits to outpatient clinics, hospital readmissions, or hospital post-discharge mortality), which could reflect poorer clinical care and inadequate management decisions, we did not observe major differences. Similarly, studies conducted in sub-Saharan African countries studying hospital readmission or post-discharge mortality have reported no differences between boys and girls [43–50].

The observed differences in referrals and admissions to hospital favouring boys, along with the finding that certain severe manifestations are less frequently observed in girls at clinical presentation, warrant further investigation. Differences in referrals and admissions to hospital may be a result of gender-based inequities in clinical evaluation and decision-making, but could also reflect the need for care and be explained by variation in signs and symptoms at presentation. The clinical impact of lower hospitalisation rates in girls remains unclear, but we did not observe more revisits to outpatient clinics or mortality among girls. In fact, mortality rates were slightly higher for boys in the entire Manhiça HDSS study area in the same period, as generally expected under conditions of equitable resource allocation and care [7,19]. Additionally, the results of this study should be interpreted in the light of analysing very large databases, where many significant relative effect measures were close to one and absolute differences in percentages between boys and girls were usually small.

One of the main strengths of our analysis is the stratification of all results in four distinct age groups, as sociocultural and biological influences on health vary with age [8,20,51]. For instance, from a biological perspective, the survival advantage in females decreases between 6–12 months after birth [6]. Also, older children could seek healthcare on their own when feeling sick and express their symptoms. In our study cohort, some results substantially differed between age groups, but differences between boys and girls remained largely consistent across all age groups. Nevertheless, we observed that girls aged 10–14 years had a higher MCBIR for outpatient clinic visits compared to boys and accounted for a larger percentage of outpatient visits, different from what was observed in other age groups. Furthermore, larger differences were observed between boys and girls in the decision to refer or admit older children to hospital from outpatient clinics.

Despite the high-quality and comprehensive data collected over 17 years, this study has some limitations. We employed an exploratory approach to examine various characteristics and outcomes, stratifying all results by age group. However, factors not considered in the analyses could act as confounders or effect modifiers and may impact the results. For instance, we did not examine how gender intersects and interacts with other dimensions of inequity (e.g. socioeconomic status, ethnicity, or disability) for which we lacked homogeneous and complete data for our entire study population [51,52]. Moreover, differences between boys and girls can arise because of both biological factors and gender-based roles. Determining the extent to which each impacts our results is challenging, as the two do not act independently but are intertwined. We acknowledge that the retrospective analysis of routine surveillance data may introduce some degree of information bias. To mitigate these, data quality control measures are established and CISM undertakes awareness and engagement activities within the community to encourage accurate reporting of events. Not all formal healthcare facilities in Manhiça district are part of the paediatric morbidity surveillance. The number of participating facilities has increased over the years, alongside demographic surveillance area expansions and an increasing number of children having an HDSS permanent identification number [21,22]. While this allows comparing outcomes for boys vs. girls, it may have introduced selection bias and limits the generalisability of findings to the entire district population and the comparison between different time periods. Moreover, we did not evaluate differences between the various outpatient clinics or specific areas within the district. We also lacked data on utilisation of informal or traditional medicine, a common resource in response to health problems in children in Manhiça district and Mozambique [53,54]. We explored differences in curative medicine but were unable to investigate disparities in preventive medicine, vaccination status, or feeding practices, which could have offered complementary information. However, previous evidence describes high levels of immunisation in Manhiça district, being similar for boys and girls [55,56].

Disaggregating data by sex or gender identity is a first critical step to address inequities in healthcare, since aggregated data sets can mask differences between boys and girls and result in the assumption that they share the same experiences and outcomes. This exploratory flagging exercise can be used as a trigger to spark further research on how and why differences arise. Although in our study population we did not observe major differences between boys and girls in most of the studied indicators of access to and provision of healthcare, similar studies in other areas of Mozambique could provide additional and complementary evidence. Further in-depth research focused on certain syndromes or aetiologies is warranted, as sex differences and gender biases during childhood can be disease-specific [6]. For example, in paediatric pneumonia, some prognostic scores for mortality incorporate sex and give a higher risk to females [57–59]. In addition, parallel qualitative research could provide valuable, context-specific insights into complex gender dynamics, offer perspectives from caregivers and healthcare providers, and help interpret the differences observed in referrals and admissions to hospital and severe manifestations at clinical presentation in Manhiça district. Lastly, the incorporation of a gender perspective and specific gender-related items in questionnaires used in the Manhiça HDSS could also aid in continuing to study potential gender disparities in children’s care and health and their underlying causes [60].

## CONCLUSIONS

The results of this study show that girls had fewer referrals and admissions to hospital in Manhiça district, but severe manifestations at clinical presentation were also less frequent in girls. Other studied indicators of healthcare access and provision were overall similar for boys and girls. Despite these generally encouraging findings, further research is needed to continue evaluating whether inherent sex differences and gender biases affect paediatric healthcare in Mozambique. Along with efforts to reduce child mortality and morbidity in the country, we must ensure sustained gender equity in the health and well-being of the paediatric population.

## Additional material


Online Supplementary Document
