# Supplementary material for: Paediatric healthcare in Manhiça district through a gender lens: a retrospective analysis of 17 years of morbidity and demographic surveillance data
Source: J Glob Health. 2025 Feb 21;15:04010. doi: 10.7189/jogh.15.04010 (PMC11843520; doi:10.7189/jogh.15.04010)
Supplement: Online Supplementary Document [file jogh-15-04010-s001.pdf]

Paediatric healthcare in Manhiça district through a gender lens: a retrospective analysis of 17 years of morbidity and demographic surveillance data

SUPPLEMENTARY MATERIAL

Núria Balanza, Aura Hunguana, Sara Ajanovic, Rosauro Varo, Justina Bramugy, Teodimiro Matsena, Tacilta Nhampossa, Dan Ouchi, Arsénio Nhacolo, Jéssica Dalsuco, Antonio Sitoe, Llorenç Quintó, Sozinho Acácio, Ariel Nhacolo, Maria Maixenchs, Khátia Munguambe, Inácio Mandomando, Pedro Aide, Francisco Saúte, Caterina Guinovart, Charfudin Sacoor, Quique Bassat

TABLE OF CONTENTS

SUPPLEMENTARY FIGURES... 2

Figure S1. Healthcare facilities covered by the morbidity surveillance of Manhiça HDSS and included in this study..2

Figure S2. Number of paediatric outpatient clinic visits and hospitalisations from 2004 to 2020 in Manhiça district 3

Figure S3. Percentage of children with severe manifestations at presentation to an outpatient clinic and upon hospitalisation in Manhiça district...4

Figure S4. Cumulative incidence of outpatient clinic revisits in children in Manhiça district....................6

Figure S5. Cumulative incidence of hospital readmissions in children in Manhiça district 7

Figure S6. Cumulative incidence of hospital post-discharge mortality in children in Manhiça district 8

Figure S7. Infant, under-5, and under-15 mortality rates in the Manhiça HDSS study area from 2004 to 2020 9

SUPPLEMENTARY TABLES 10

Table S1. Definitions used for severe manifestations...10

Table S2. Minimum community-based incidence rates of outpatient clinic visits and hospitalisations in children living in the Manhiça HDSS study area from 2004 to 2020..10

Table S3. Girls-to-boys odds ratios of outcomes of paediatric outpatient clinic visits and hospitalisations in Manhiça district 11

Table S4. Length of hospital stay in children admitted to Manhiça District Hospital 12

Table S5. Diagnostic tests and treatments in children admitted to Manhiça District Hospital 13

Table S6. Time to death in children admitted to Manhiça District Hospital who died in-hospital 14

Table S7. Hazard ratios and subdistribution hazard ratios for post-discharge outcomes in children after an outpatient clinic visit and after hospitalisation in Manhiça district 15

SUPPLEMENTARY FIGURES

Figure S1. Healthcare facilities covered by the morbidity surveillance of Manhiça HDSS and included in this study

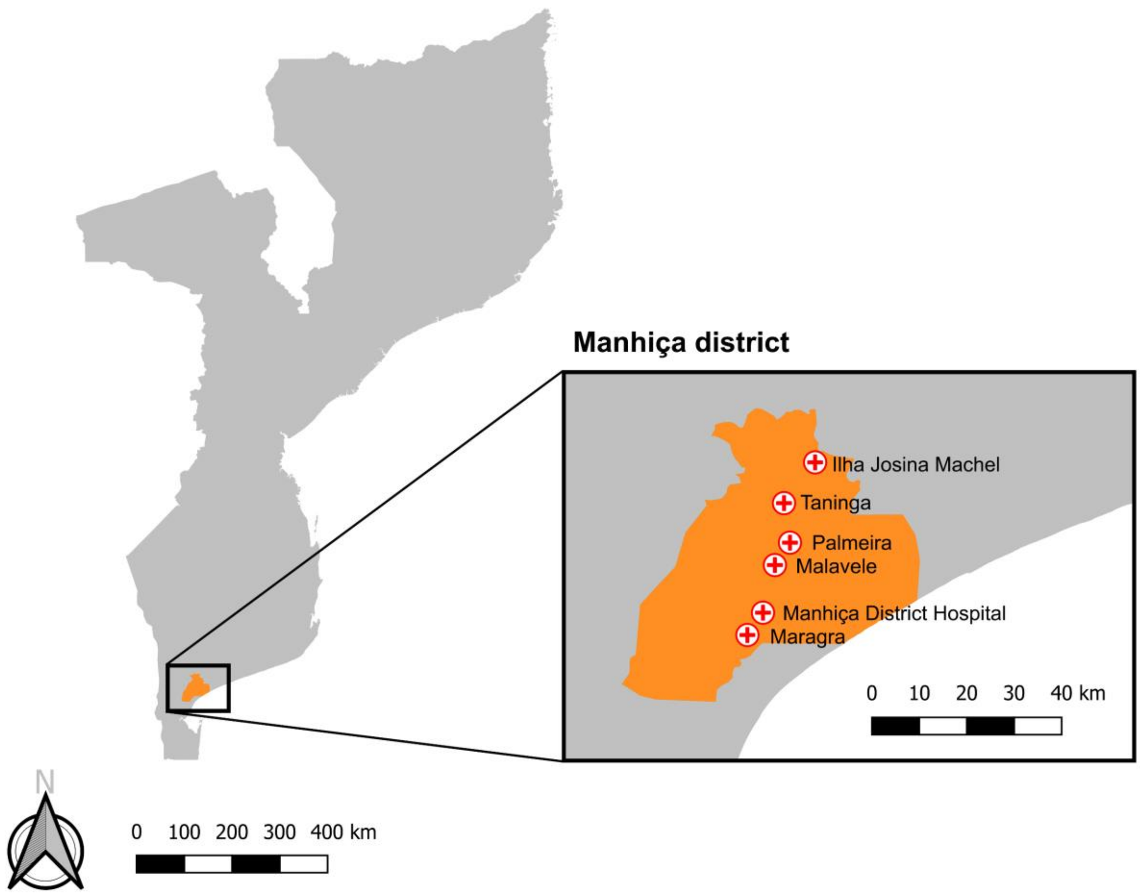

Figure S2. Number of paediatric outpatient clinic visits and hospitalisations from 2004 to 2020 in Manhiça district

A) Visits to six outpatient clinics

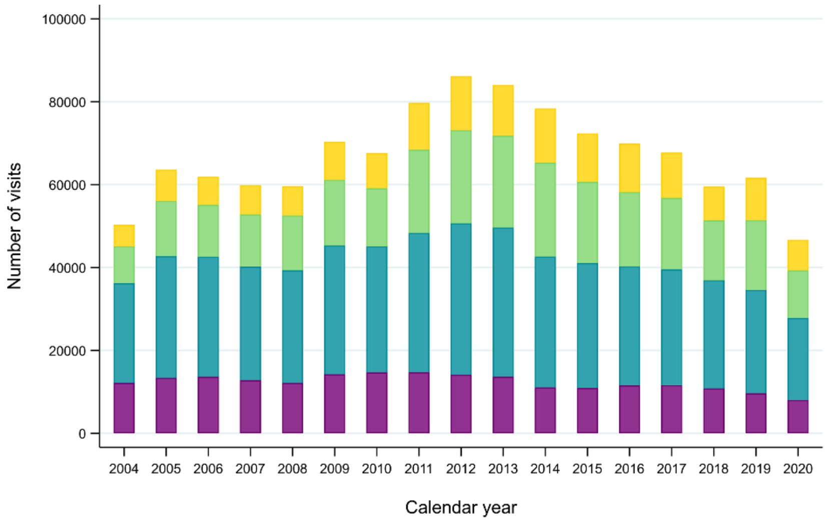

B) Admissions to Manhiça District Hospital

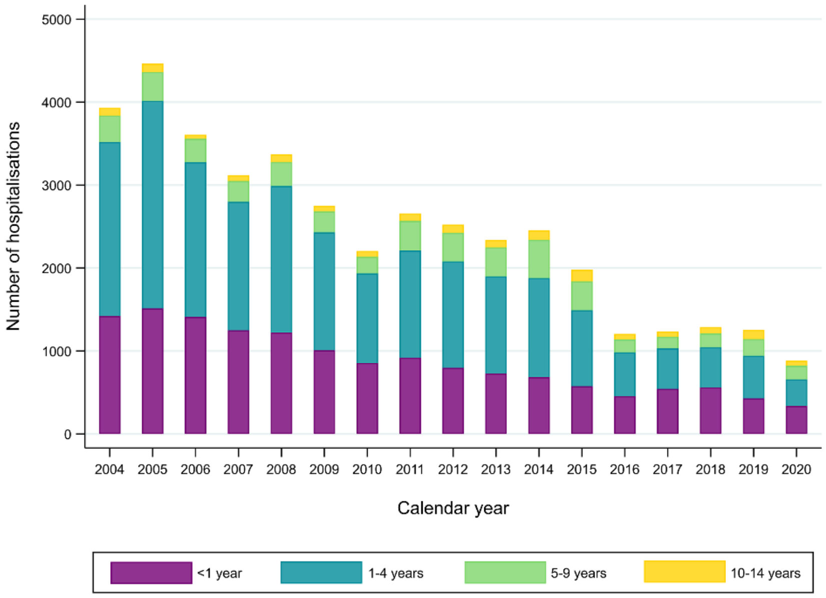

Data on outpatient clinic visits were collected from: Manhiça District Hospital (2004-2020), Maragra (2004-2020), Ilha Josina Machel (2004-2020), Taninga (2005-2020), Palmeira (2009-2020), and Malavele (2009-2020) outpatient clinics.

Figure S3. Percentage of children with severe manifestations at presentation to an outpatient clinic and upon hospitalisation in Manhiça district

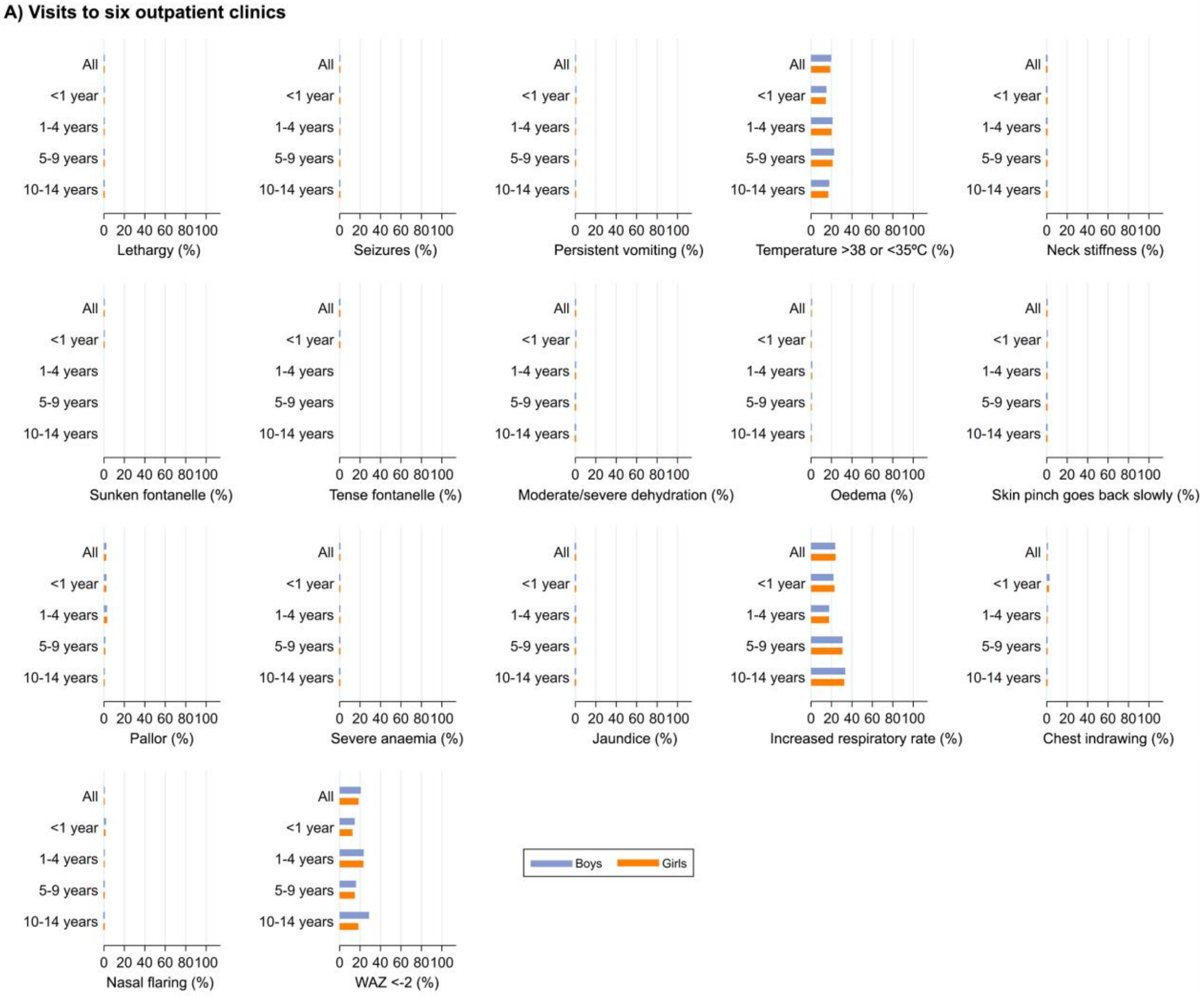

B) Admissions to Manhiça District Hospital

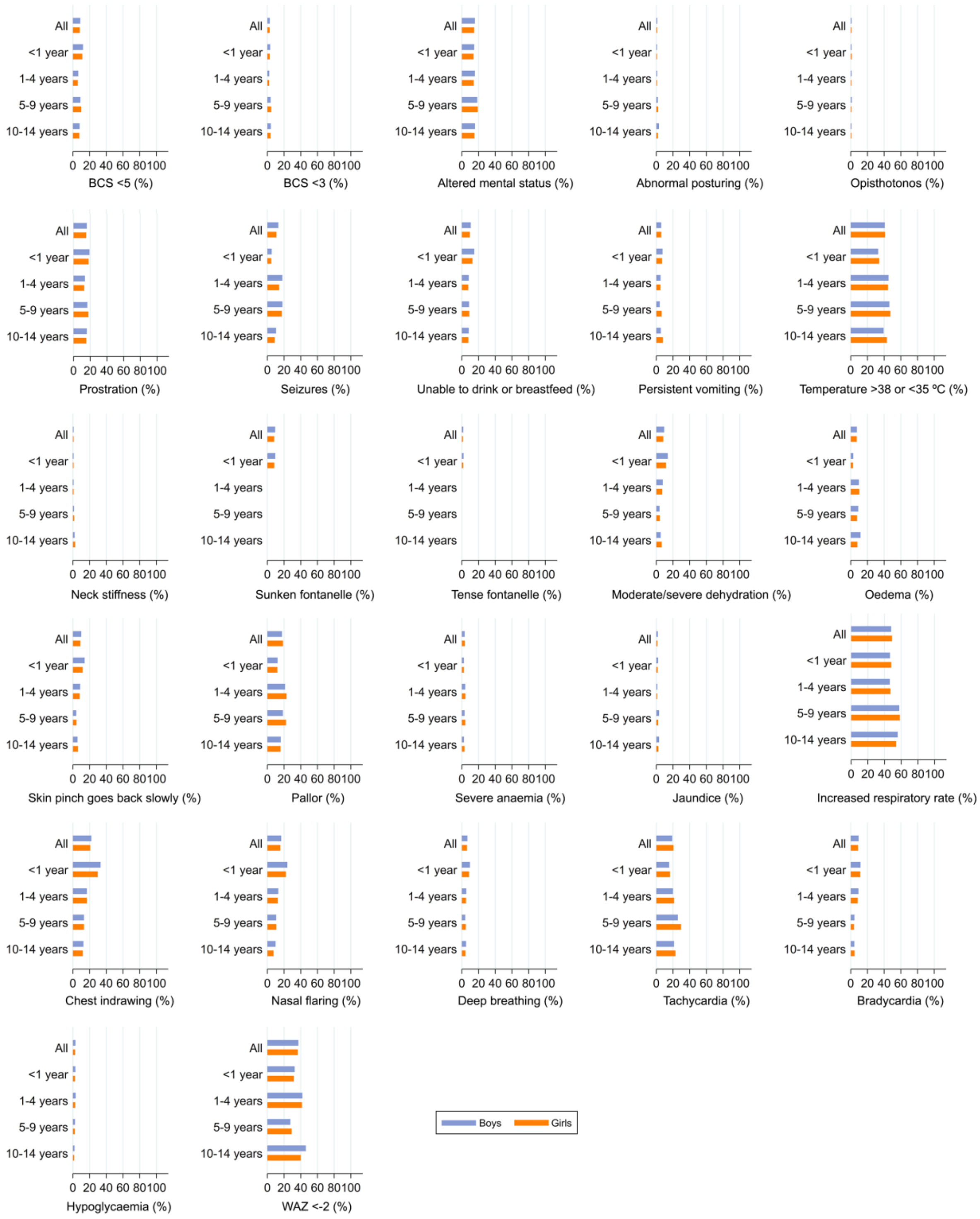

Definitions used for severe manifestations can be found in Table S1. Sunken or tense fontanelle was calculated only in children aged <1 year. Missing data in all variables is <5%, except for sunken/tense fontanelle (5% in outpatient clinic visits, 8% in hospitalisations), severe anaemia (45% in outpatient clinic visits, 9% in hospitalisations), and hypoglycaemia (9% in hospitalisations). Abbreviations: BCS=Blantyre Coma Scale, WAZ=weight-for-age z-score.

Figure S4. Cumulative incidence of outpatient clinic revisits in children in Manhiça district

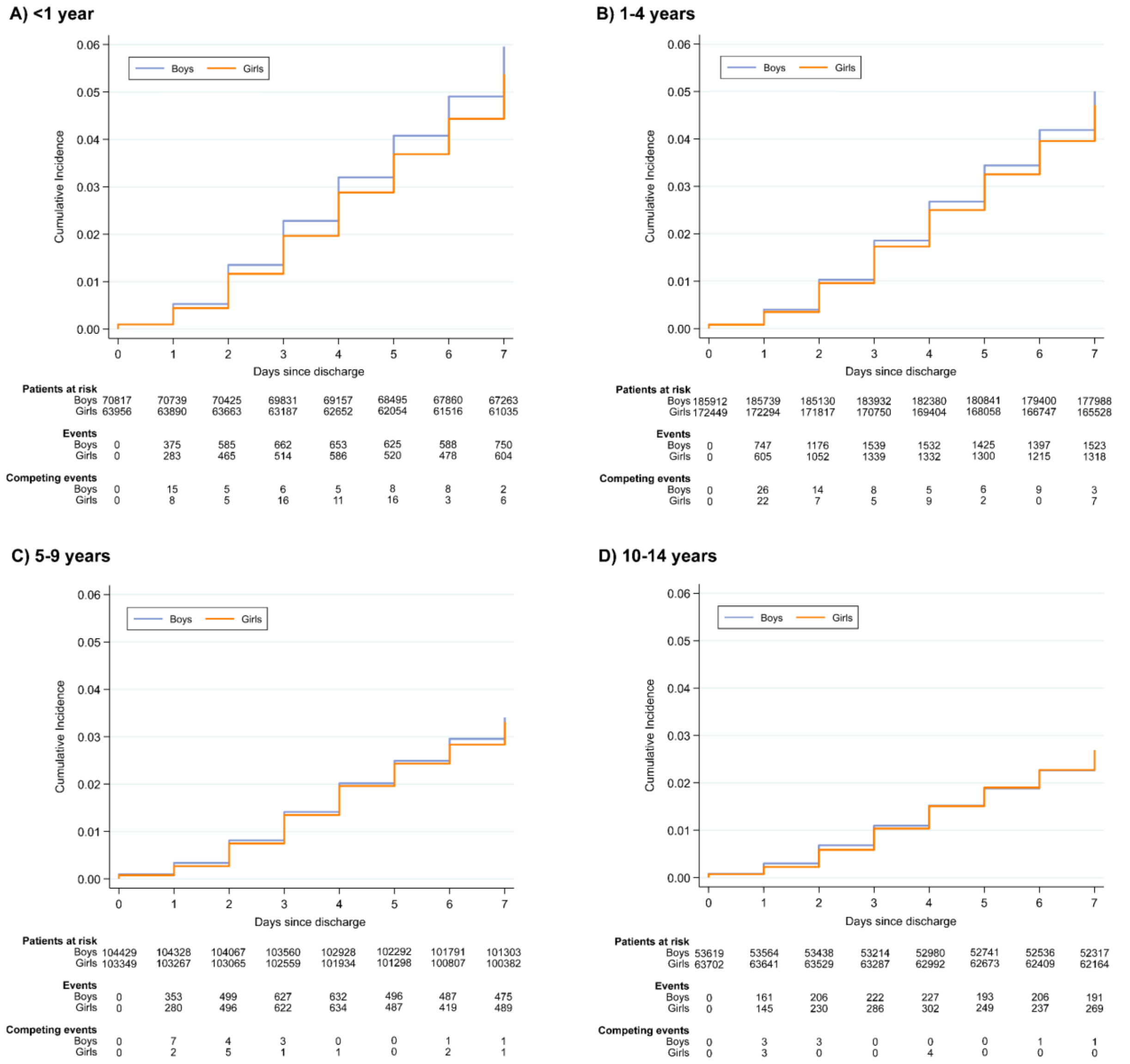

Index visits were defined as those in which children were discharged home or absconded, and death was treated as a competing event.

Figure S5. Cumulative incidence of hospital readmissions in children in Manhiça district

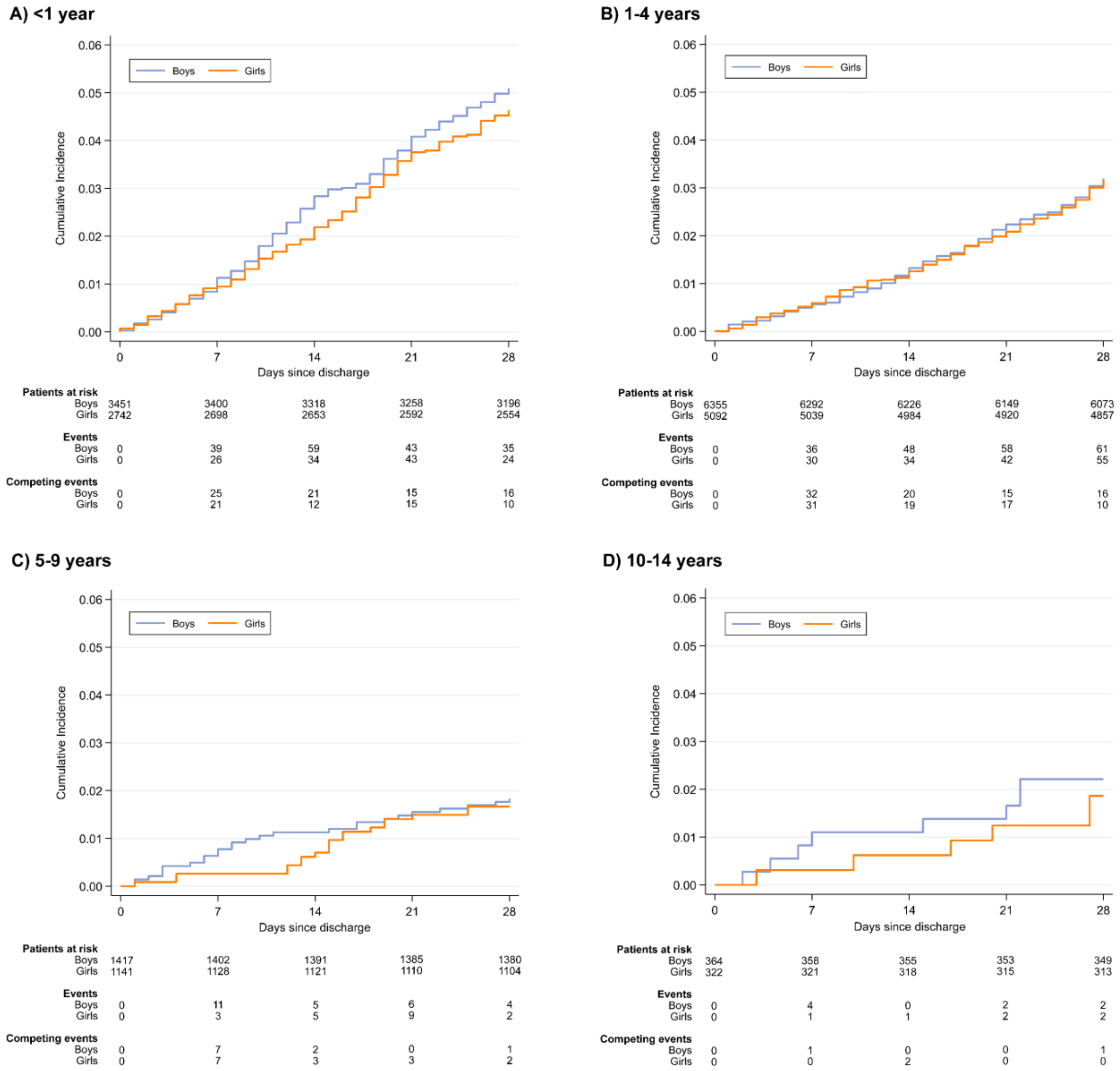

Index hospitalisations were defined as those in which children were discharged home or absconded, and death was treated as a competing event.

Figure S6. Cumulative incidence of hospital post-discharge mortality in children in Manhiça district

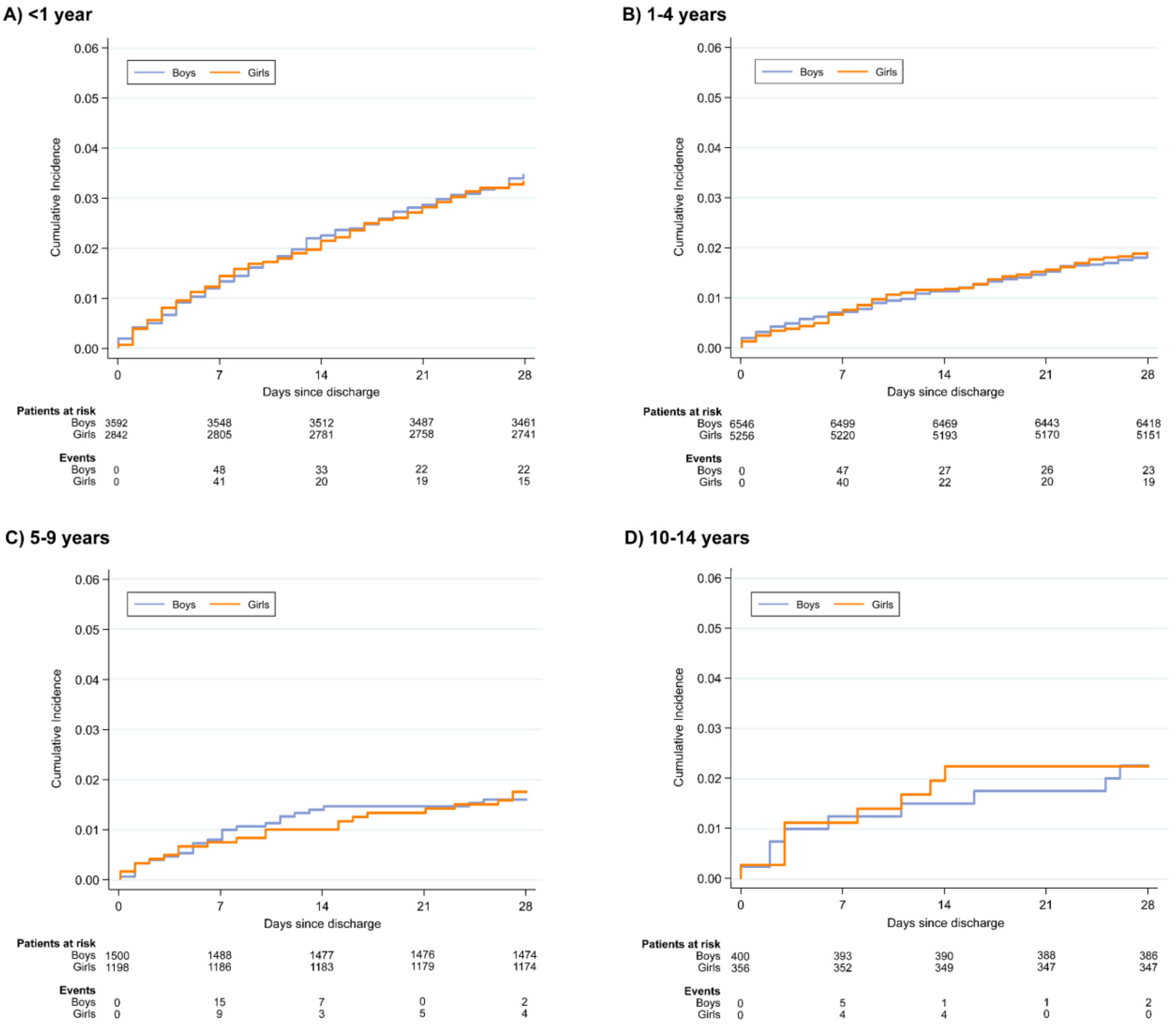

Index hospitalisations were defined as those with a known outcome different from in-hospital death.

Figure S7. Infant, under-5, and under-15 mortality rates in the Manhiça HDSS study area from 2004 to 2020

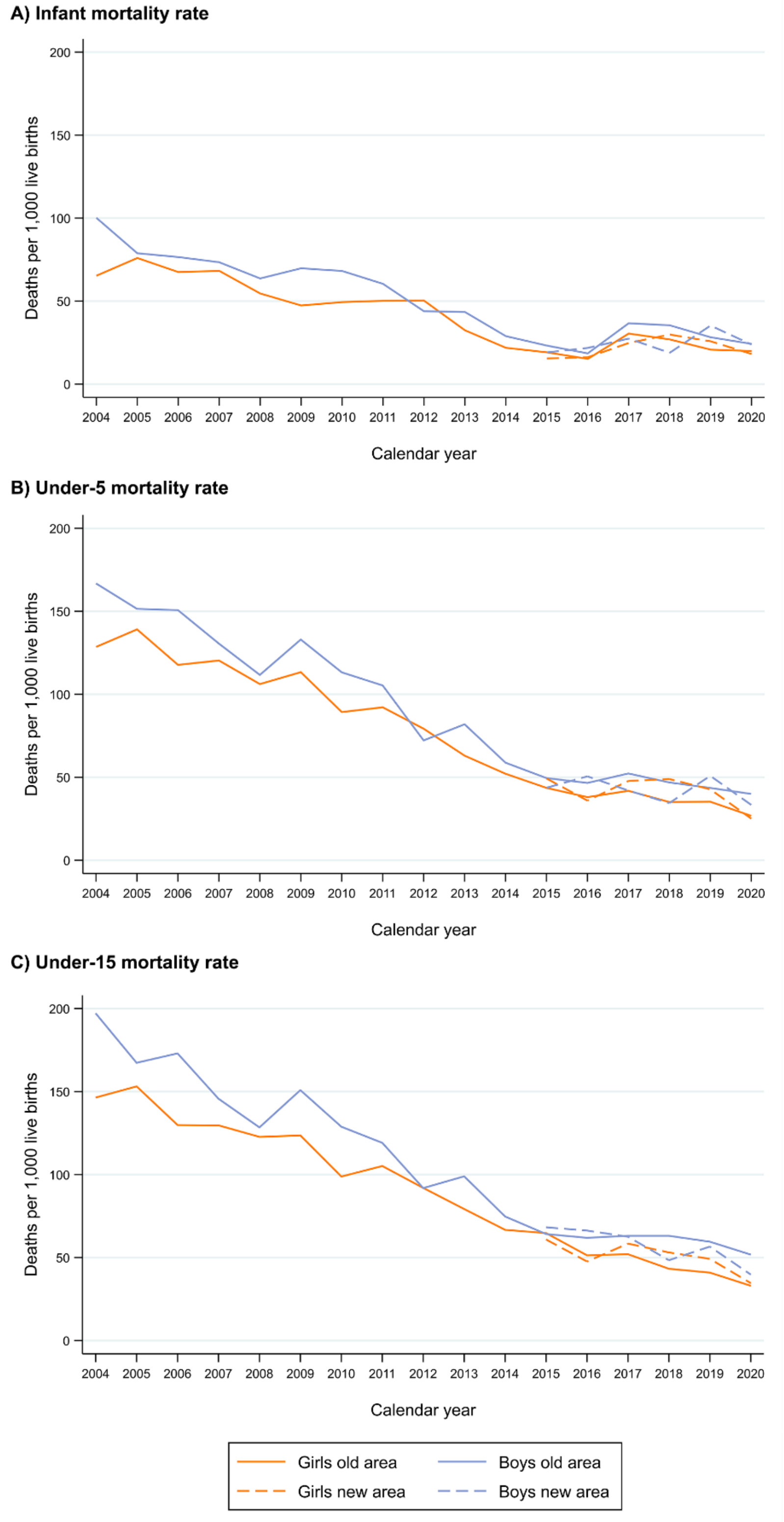

Mortality rates were computed as the number of deaths (in infants, children younger than five years, or children younger than fifteen years) in each calendar year divided by the total number of live births that occurred in the same year, expressed per thousand live births. Data are shown stratified by old and new Manhiça HDSS demographic surveillance areas.

SUPPLEMENTARY TABLES

Table S1. Definitions used for severe manifestations

Severe manifestation	Definition
Persistent vomiting	Reported vomiting for ≥2 days.
Prostration	Inability to sit unaided or look for a mother's breast/feed in children who are not yet able to sit.
Severe anaemia	Haematocrit <25% in children ≤28 days and haematocrit <15% in children >28 days.
Increased respiratory rate	≥60 breaths/min in children aged <2 months, ≥50 breaths/min in children aged 2–11 months, ≥40 breaths/min in children aged 12–59 months, ≥30 breaths/min in children aged 5–12 years, and ≥20 breaths/min in children aged 13–14 years.
Tachycardia and bradycardia	Above or under the normal ranges of: 100–180 beats/min in children aged <1 month, 100–160 beats/min in children aged 1–11 months, 90–150 beats/min in children aged 1–2 years, 80–140 beats/min in children aged 3–5 years, 70–120 beats/min in children aged 6–12 years, and 60–100 beats/min in children aged 13–14 years.
Hypoglycaemia	Glycemia <2.5 mmol/L.
Weight-for-age z-score	Calculated using the LMS method and the 2000 US Centers for Disease Control and Prevention Growth Reference data (Vidmar *et al.*, 2013).

Table S2. Minimum community-based incidence rates of outpatient clinic visits and hospitalisations in children living in the Manhiça HDSS study area from 2004 to 2020

	Visits to six outpatient clinics		Admissions to Manhiça District Hospital	
	MCBIR per 1,000 child-years at risk	IRR (95% CI)	MCBIR per 1,000 child-years at risk	IRR (95% CI)
**All**				
Boys	906.05	1	26.18	1
Girls	878.44	0.97 (0.96-0.98)	21.10	0.81 (0.79-0.84)
**<1 year**				
Boys	2282.57	1	113.43	1
Girls	2091.86	0.96 (0.95-0.96)	91.57	0.82 (0.78-0.86)
**1-4 years**				
Boys	1463.20	1	50.22	1
Girls	1364.72	0.96 (0.96-0.97)	41.00	0.83 (0.80-0.87)
**5-9 years**				
Boys	643.82	1	9.24	1
Girls	632.80	0.97 (0.96-0.99)	7.37	0.80 (0.74-0.86)
**10-14 years**				
Boys	370.14	1	2.86	1
Girls	439.14	1.09 (1.07-1.11)	2.50	0.85 (0.73- 0.98)

IRRs calculated by fitting mixed-effects negative binomial regression models, with individual as a random effect. Abbreviations: CI=confidence interval, IRRs=incidence rate ratios, MCBIR = minimum community-based incidence rate.

Table S3. Girls-to-boys odds ratios of outcomes of paediatric outpatient clinic visits and hospitalisations in Manhiça district

Visits to five outpatient clinics (excluding the one at Manhiça District Hospital):															
	**All**			**<1 year**			**1-4 years**			**5-9 years**			**10-14 years**		
	**Boys, N (%)**	**Girls, N (%)**	**OR (95% CI)**	**Boys, N (%)**	**Girls, N (%)**	**OR (95% CI)**	**Boys, N (%)**	**Girls, N (%)**	**OR (95% CI)**	**Boys, N (%)**	**Girls, N (%)**	**OR (95% CI)**	**Boys, N (%)**	**Girls, N (%)**	**OR (95% CI)**
**Discharge home**	314,877 (97.9%)	310,820 (98.3%)	1	52,791 (96.7%)	48,221 (96.7%)	1	137,294 (97.7%)	128,273 (97.9%)	1	79,716 (98.6%)	80,397 (99.1%)	1	45,076 (99.0%)	53,929 (99.4%)	1
**Absconder**	844 (0.3%)	825 (0.3%)	0.99 (0.90-1.09)	199 (0.4%)	193 (0.4%)	1.06 (0.87-1.29)	329 (0.2%)	354 (0.3%)	1.15 (0.99-1.34)	182 (0.2%)	153 (0.2%)	0.83 (0.67-1.03)	134 (0.3%)	125 (0.2%)	0.78 (0.61-1.00)
**Transfer**	5,793 (1.8%)	4,707 (1.5%)	0.82 (0.79-0.86)	1,604 (2.9%)	1,450 (2.9%)	0.99 (0.92-1.06)	2,918 (2.1%)	2,467 (1.9%)	0.90 (0.86-0.96)	933 (1.2%)	592 (0.7%)	0.63 (0.57-0.70)	338 (0.7%)	198 (0.4%)	0.49 (0.41-0.58)
Visits to the outpatient clinic at Manhiça District Hospital:															
	**All**			**<1 year**			**1-4 years**			**5-9 years**			**10-14 years**		
	**Boys, N (%)**	**Girls, N (%)**	**OR (95% CI)**	**Boys, N (%)**	**Girls, N (%)**	**OR (95% CI)**	**Boys, N (%)**	**Girls, N (%)**	**OR (95% CI)**	**Boys, N (%)**	**Girls, N (%)**	**OR (95% CI)**	**Boys, N (%)**	**Girls, N (%)**	**OR (95% CI)**
**Discharge home**	233,701 (91.1%)	224,428 (92.3%)	1	47,435 (85.7%)	42,455 (86.7%)	1	104,651 (90.5%)	96,550 (91.6%)	1	54,218 (95.1%)	53,361 (95.8%)	1	27,397 (96.4%)	32,062 (97.1%)	1
**Absconder**	1,380 (0.5%)	1,220 (0.5%)	0.92 (0.85-0.99)	402 (0.7%)	331 (0.7%)	0.92 (0.79-1.06)	564 (0.5%)	485 (0.5%)	0.93 (0.83-1.05)	244 (0.4%)	224 (0.4%)	0.93 (0.78-1.12)	170 (0.6%)	180 (0.6%)	0.90 (0.73-1.12)
**Transfer**	788 (0.3%)	598 (0.3%)	0.79 (0.71-0.88)	221 (0.4%)	169 (0.4%)	0.85 (0.70-1.04)	294 (0.3%)	242 (0.2%)	0.89 (0.75-1.06)	187 (0.3%)	116 (0.2%)	0.63 (0.50-0.79)	86 (0.3%)	71 (0.2%)	0.71 (0.51-0.97)
**Admission**	20,509 (8.0%)	16,829 (6.9%)	0.85 (0.84-0.87)	7,293 (13.2%)	5,992 (12.2%)	0.92 (0.88-0.95)	10,104 (8.7%)	8,156 (7.7%)	0.87 (0.85-0.90)	2,338 (4.1%)	1,975 (3.6%)	0.86 (0.81-0.91)	774 (2.7%)	706 (2.1%)	0.78 (0.70-0.86)
**Death**	57 (0.02%)	53 (0.02%)	0.97 (0.67-1.41)	20 (0.04%)	19 (0.04%)	1.06 (0.57-1.99)	20 (0.02%)	26 (0.02%)	1.41 (0.79-2.52)	11 (0.02%)	7 (0.01%)	0.65 (0.25-1.67)	6 (0.02%)	1 (<0.01%)	0.14 (0.02-1.18)
Admissions to Manhiça District Hospital:															
	**All**			**<1 year**			**1-4 years**			**5-9 years**			**10-14 years**		
	**Boys, N (%)**	**Girls, N (%)**	**OR (95% CI)**	**Boys, N (%)**	**Girls, N (%)**	**OR (95% CI)**	**Boys, N (%)**	**Girls, N (%)**	**OR (95% CI)**	**Boys, N (%)**	**Girls, N (%)**	**OR (95% CI)**	**Boys, N (%)**	**Girls, N (%)**	**OR (95% CI)**
**Discharge home**	19,773 (88.0%)	16,214 (87.8%)	1	6,819 (84.9%)	5,573 (84.8%)	1	10,082 (90.1%)	8,174 (89.6%)	1	2,228 (89.9%)	1,869 (89.8%)	1	644 (84.7%)	598 (86.8%)	1
**Absconder**	856 (3.8%)	756 (4.1%)	1.08 (0.97-1.19)	356 (4.4%)	334 (5.1%)	1.15 (0.98-1.34)	433 (3.9%)	365 (4.0%)	1.04 (0.90-1.20)	49 (2.0%)	46 (2.2%)	1.12 (0.74-1.68)	18 (2.4%)	11 (1.6%)	0.66 (0.31-1.40)
**Transfer**	998 (4.4%)	786 (4.3%)	0.96 (0.87-1.06)	410 (5.1%)	305 (4.6%)	0.91 (0.78-1.06)	361 (3.2%)	294 (3.2%)	1.00 (0.86-1.17)	147 (5.9%)	118 (5.7%)	0.96 (0.75-1.23)	80 (10.6%)	69 (10.0%)	0.93 (0.66-1.31)
**Death**	841 (3.7%)	706 (3.8%)	1.02 (0.92-1.13)	452 (5.6%)	358 (5.5%)	0.97 (0.84-1.12)	317 (2.8%)	288 (3.2%)	1.12 (0.95-1.32)	54 (2.2%)	49 (2.4%)	1.08 (0.73-1.60)	18 (2.4%)	11 (1.6%)	0.66 (0.31-1.40)

All girls-to-boys odd ratios were estimated from fitting univariable multinomial logistic regression models. Abbreviations: CI=confidence interval, OR=odds ratio.

Table S4. Length of hospital stay in children admitted to Manhiça District Hospital

	All children		Children discharged home	
	**N**	**Median days (IQR)**	**N**	**Median days (IQR)**
**All**				
Boys	22,474	3 (2-5)	19,646	3 (2-5)
Girls	18,465	3 (2-5)	16,104	3 (2-5)
**<1 year**				
Boys	8,022	4 (2-6)	6,764	4 (3-6)
Girls	6,545	4 (2-6)	5,524	4 (3-6)
**1-4 years**				
Boys	11,198	3 (2-5)	10,033	3 (2-5)
Girls	9,147	3 (2-5)	8,134	3 (2-5)
**5-9 years**				
Boys	2,491	3 (2-4)	2,210	3 (2-4)
Girls	2,082	3 (2-5)	1,851	3 (2-5)
**10-14 years**				
Boys	763	3 (2-5)	639	3 (2-5)
Girls	691	3 (2-5)	595	3 (2-5)

All standardized mean differences in length of hospital stay between boys and girls are <0.1. Abbreviations: IQR=interquartile range.

Table S5. Diagnostic tests and treatments in children admitted to Manhiça District Hospital

	All		<1 year		1-4 years		5-9 years		10-14 years	
	Boys, (%) (N=22,653)	Girls, (%) (N=18,625)	Boys, (%) (N=8,096)	Girls, (%) (N=6,619)	Boys, (%) (N=11,271)	Girls, (%) (N=9,203)	Boys, (%) (N=2,516)	Girls, (%) (N=2,105)	Boys, (%) (N=770)	Girls, (%) (N=698)
**Antibiotics**	68.5%	66.8%	82.6%	80.5%	61.5%	60.0%	56.5%	56.1%	63.3%	59.5%
**Antimalarials**	44.2%	46.4%	26.4%	29.4%	54.2%	55.9%	56.9%	58.2%	44.8%	48.2%
**Blood culture**	81.9%	81.2%	93.0%	93.3%	80.6%	79.4%	60.7%	60.6%	53.6%	51.3%
**Blood smear**	96.3%	96.9%	94.4%	95.1%	97.8%	98.1%	96.3%	97.7%	94.1%	94.0%
**Blood transfusion**	6.6%	7.4%	4.5%	4.9%	8.4%	9.3%	5.9%	8.0%	4.7%	4.9%
**Chest X-ray**	27.3%	27.0%	30.3%	28.1%	26.8%	27.3%	20.8%	23.4%	24.8%	24.6%
**HIV test**	24.0%	23.5%	18.7%	17.2%	26.6%	27.0%	28.1%	27.0%	29.3%	26.9%
**Lumbar puncture**	13.6%	12.3%	14.7%	14.4%	12.9%	10.9%	15.1%	12.9%	9.2%	8.2%

Missing data in all variables is <2%.

Table S6. Time to death in children admitted to Manhiça District Hospital who died in-hospital

	All children	
	**N**	**Median days (IQR)**
**All**		
Boys	838	2 (1-5)
Girls	703	2 (1-5)
**<1 year**		
Boys	450	2 (1-5)
Girls	356	2 (1-5)
**1-4 years**		
Boys	316	2 (1-7)
Girls	287	2 (1-6)
**5-9 years**		
Boys	54	1 (0-3)
Girls	49	1 (0-3)
**10-14 years**		
Boys	18	0.5 (0-2)
Girls	11	0 (0-5)

All standardized mean differences in length of hospital stay between boys and girls are <0.2. Abbreviations: IQR=interquartile range.

Table S7. Hazard ratios and subdistribution hazard ratios for post-discharge outcomes in children after an outpatient clinic visit and after hospitalisation in Manhiça district

	Revisits to outpatient clinics within 7 days, SHR (95% CI)	Hospital readmissions within 28 days, SHR (95% CI)	Hospital post-discharge mortality within 28 days, HR (95% CI)
**All**			
Boys	1	1	1
Girls	0.93 (0.90-0.95)	0.94 (0.81-1.10)	1.00 (0.84-1.19)
**<1 year**			
Boys	1	1	1
Girls	0.90 (0.86-0.94)	0.91 (0.72-1.14)	0.96 (0.74-1.25)
**1-4 years**			
Boys	1	1	1
Girls	0.94 (0.91-0.97)	0.99 (0.80-1.22)	1.02 (0.79-1.33)
**5-9 years**			
Boys	1	1	1
Girls	0.97 (0.92-1.02)	0.91 (0.50-1.64)	1.10 (0.61-1.96)
**10-14 years**			
Boys	1	1	1
Girls	1.03 (0.95-1.11)	0.84 (0.29-2.41)	0.99 (0.39-2.56)

For revisits to outpatient clinics and hospital readmissions, index visits or admissions were defined as those in which children were either discharged home or absconded. Fine-Gray competing risk regression models were used to calculate SHRs for revisits to outpatient clinics and for hospital readmissions, considering death as a competing event. For hospital post-discharge mortality, index hospitalisations were defined as those with a known outcome different from in-hospital death. Cox proportional hazards regression models were used to calculate HRs for hospital post-discharge mortality. In all analyses, children who migrated outside the Manhiça HDSS study area before the end of the follow-up period were censored at that moment. Standard errors were adjusted for within-child correlation. Abbreviations: CI=confidence interval, HR=hazard ratio, SHR=subdistribution hazard ratio.
